# Size Control and Growth Process Study of Au@Cu_2_O Particles

**DOI:** 10.1186/s11671-016-1603-6

**Published:** 2016-09-08

**Authors:** Yuyuan Wang, Min Zheng, Shengnan Liu, Zuoshan Wang

**Affiliations:** 1 College of Chemistry, Chemical Engineering and Materials Science, Soochow University, Soochow, 215123 China; 2 College of Textile and Clothing Engineering, Soochow University, Soochow, 215123 China

**Keywords:** Core-shell structure, Triangular nanoplate, Size control, Visible photocatalyst

## Abstract

Au@Cu_2_O cuboctahedron with gold triangular nanoplate core and Cu_2_O shell was synthesized by a chemical method. X-ray diffraction (XRD) and transmission electron microscopy (TEM) tests demonstrated that the as-synthesis samples were consisted of gold triangular nanoplate core and Cu_2_O shell, and both of them were in good crystallization. The effective size control of the particles could be realized by controlling the amount of Au cores added in the synthetic process and Au@Cu_2_O particles with different shell thickness could be synthesized. The decrease of Cu_2_O shell thickness had a great difference in the optical performance, including blue shift of the resonant peaks and enhanced absorption intensity. The growth process from rough sheet structure to cuboctahedron was also explored. The results of photocatalytic degradation experiment showed that Au@Cu_2_O particles showed much better photocatalytic performance than that of pure Cu_2_O. The improved photocatalytic property of the Au@Cu_2_O particles was attributed to the comprehensive effect of the enhanced visible-light absorption and high separation rate of electron-hole pairs.

**Electronic supplementary material:**

The online version of this article (doi:10.1186/s11671-016-1603-6) contains supplementary material, which is available to authorized users.

## Background

Photocatalyst can take advantage of solar power to generate redox couple for the degradation of organic pollutant. In recent years, many significant achievements about photocatalyst have been made [1–6]; however, photocatalyst still faces several problems which include low photocatalytic efficiency and unsuitable light absorption region. The former is mainly caused by the high recombination rate of electron-hole pairs, such as Bi_2_S_3_ [7], while the latter is attributed to the wide band gap, such as TiO_2_ [8] and ZnO [9]. The two disadvantages have seriously limited photocatalyst’s further applications. Thus, how to broaden the visible-light absorption range or promote electron-hole pairs separating has been an effective direction to push the research forward. The design of core-shell structure with suitable bandgap semiconductors seems to be a viable way to figure out these problems. In previous reports, Cu_2_O has been proved to be an ideal visible photocatalyst for its large light absorption coefficient, high refractive index and low cost [2, 10, 11]. However, the photocatalytic performance of pure Cu_2_O is far from satisfaction due to its wide size distribution and the high recombination rate of electron-hole pairs [12–14]. To get over these difficulties, modification of Cu_2_O with plasmonic nanoparticles seems to be an efficient way [2, 5, 15–21].

Plasmonic nanoparticles, especially noble metal, have strong localized surface plasmon resonance (LSPR) in the visible spectrum range, which arouses extensive interests into the preparation of high-efficiency plasmonic photocatalysts. Their LSPR can be further adjusted by controlling sizes, morphologies and surrounding dielectric environment. Compared with noble metal with other morphologies, triangular nanoplate (TN) has more efficient charge separation and red-shifted optical coefficient peak due to the integrated effect of its two-dimensional structure, sharp corners and anisotropy [16]. These properties can improve the photocatalytic performance enormously. Thus, Au TN can be an excellent candidate for the modification of Cu_2_O. The heterogeneous nucleation of Cu_2_O not only helps overcome the shortcomings of large size and wide size distribution but also can tune its light absorption scope.

Reports about the construction of Au@Cu_2_O core-shell structure were abundant [5, 17–19], and various kinds of Au cores have been involved, such as sphere [15], rod [17], cube [5] and octahedron [18]. However, as far as we know, few of them referred to the two-dimensional nanoplate core. Huang [22] and Mirkin [23] have prepared core-shell structure with triangular nanoplates as core. In their work, the core-shell heterostructures were successfully synthesized with good crystallinity. However, the morphology of the heterostructures only exhibited sheet structure, and the role of Au TN and the properties influenced by particle size have not been explored. Inspired by their work, we have designed a core-shell structure with triangular Au nanoplate core and Cu_2_O shell. Moreover, the shell thickness of the Au@Cu_2_O cuboctahedrons could be adjusted from 159 to 53 nm while symmetry and morphology of the cuboctahedrons were unaltered. Compared with the pure Cu_2_O, the photocatalytic property of the Au@Cu_2_O particles was improved greatly. The role of the Au TN, growth process, size control, optical properties and photocatalytic activity of the samples were analyzed in details.

## Methods

### Synthesis of gold TN

Gold TN was synthesized by the method reported with little modification [24]. Briefly, 8 ml of 0.1 M hexadecyltrimethylammonium chloride (CTAC) was first added into a 100-ml conical flask with 40 ml of deionized water in it, and then 375 μl of 0.01 M KI was added. After the injection of 400 μl of 25.4 mM HAuCl_4_ and 100 μl of 0.1 M NaOH, the solution began to show light yellowish color. Then 400 μl of 64 mM ascorbic acid (AA) was added, and once AA was injected, the yellowish solution turned to colorless. At last, 50 μl of 0.1 M NaOH was added and stirred for several seconds. Then the reaction was left for 10 min without disturbance for complete crystalline growth. As the reaction proceeded, we could see the colorless solution turned to pink, then purple and at last blue. The triangular plates were detached and washed by centrifugation with deionized water. Then the Au TNs were attenuated into 3.1 ml.

### Synthesis of Au@Cu_2_O Cuboctahedron

Au@Cu_2_O cuboctahedron was prepared by the method reported with little modification [17]. In a 20-ml beaker, (8.9-*x*) ml of deionized H_2_O, 300 μl of 0.1 M CuCl_2_ solution, and 0.087 g of sodium dodecylsulfate (SDS) were added in sequence. Then *x* ml of the gold TN solution and 300 μl of 1 M NaOH solution were injected. Here, *x* stood for the volume of gold TN solution added. To get different shell thickness, the concrete values of *x* were set as 0, 0.1, 0.2, 0.4, 0.8, and 1.6, respectively. The total solution volume was kept at 10 ml. After stirring for 10 min, 500 μl of 0.2 M hydroxylamine hydrochloride (NH_2_OH·HCl) was added dropwise into the reaction solution. Two hours later after aging was finished, the product was gathered by centrifugation. The samples were designated as *y*-Au@Cu_2_O where *y* represented the shell thickness of the samples. Then the collection was washed with ethanol and deionized water several times to get rid of impurities ion and surfactant.

### Characterization of the Samples

The crystalline phase of products prepared was identified by X-ray diffraction (XRD) with Cu-Ka radiation. Scanning electron microscopy (SEM) was taken on a S4800 SEM instrument. Transmission electron microscopy (TEM) and high-resolution transmission electron microscopy (HRTEM) (Hitachi H600A, Tokyo, Japan) were utilized to characterize structure. UV-3600 UV-vis-NIR spectrophotometer was used to characterize the UV-vis diffuse reflectance spectra. The specific surface areas of the samples were tested from nitrogen adsorption-desorption isotherms on a Micromeritics Tristar 3020 system.

### Photocatalytic Activity

The photocatalytic evaluation of the samples was carried out by dispersing 0.01 g of the product for the degradation of 100 ml MO solution (10 mg/l). A 500 W xenon lamp was chosen as visible-light source. Then the solution was stirred without light for 20 min to make sure that MO solution had got absorption equilibrium state. After every 20 min, 3–4 ml solution was taken out and centrifuged. UV-vis spectrophotometer was used to measure the product. The residual portion of MO could be worked out by the formula *η* = *C*/*C*_*0*_, where the original concentration and subsequent concentration of MO solution were respectively denoted as *C*_*0*_ and *C*.

### Reactive Species Trapping Experiments

Five millimolar triethanolamine (TEOA), isopropanol (IPA), and ascorbic acid (AA) were added to the MO solution as h^+^ scavenger, ·OH scavenger and ·O_2_^−^ scavenger, respectively. And the concentration of MO was measured after irradiation for 40 min. The degradation percentages of MO solution were calculated by the formula (C_0_ − C)/C_0_.

## Results and Discussion

### Phase and Crystal Structure Analysis

**Fig. 1:**
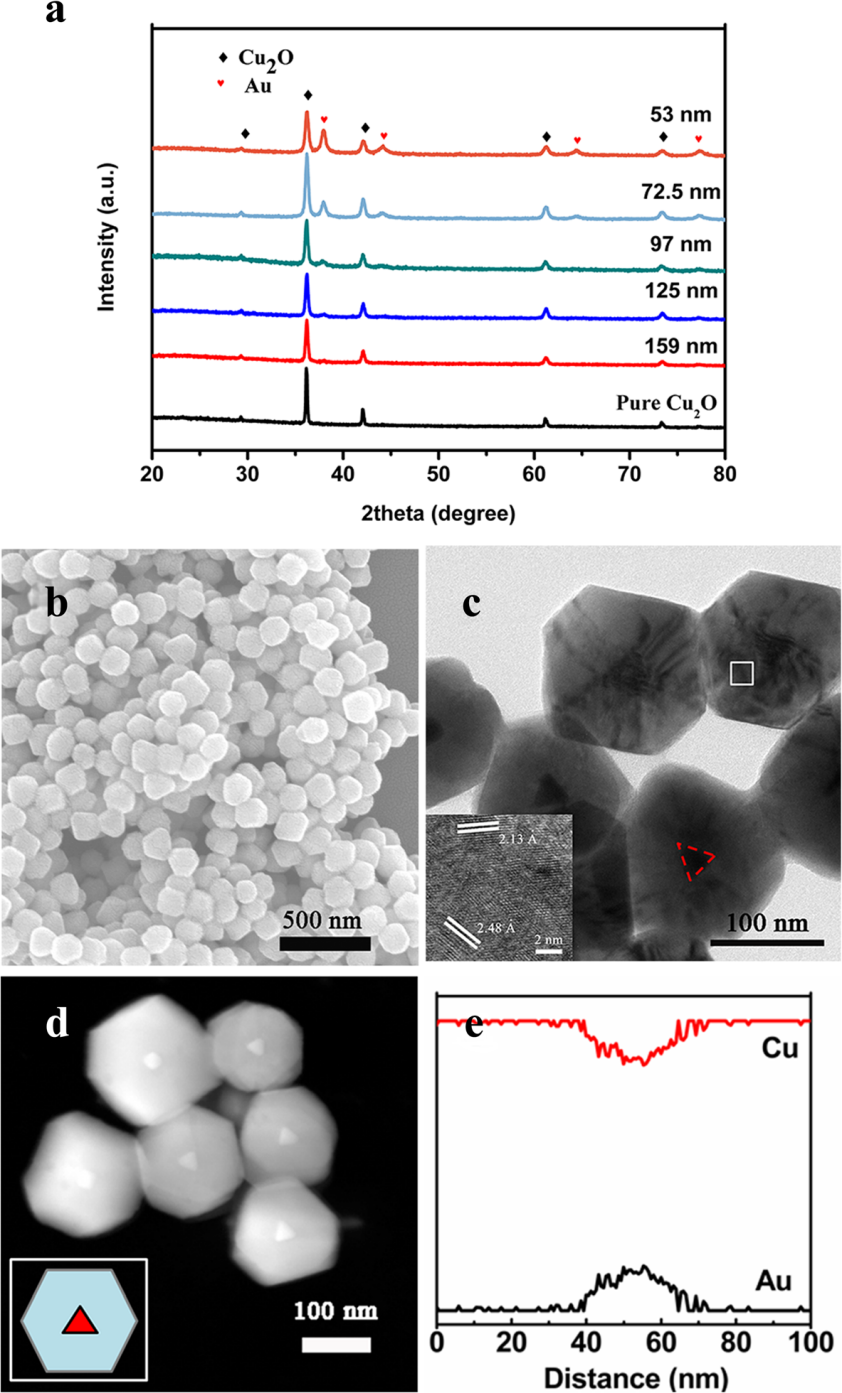
**a** XRD patterns, **b** SEM, **c** TEM, **d** STEM, and **e** cross-sectional compositional line profiles images of the synthesized samples. The inset in **c** is the HRTEM image of selected area

The XRD patterns of the samples were showed in Fig. 1a. The results distinctly demonstrated that there were two sets of diffraction data, which accorded well with the cubic Au (JCPDS no. 89-3697) and cubic phase Cu_2_O (JCPDS no. 05-0667). This indicated the synthesis of Au and Cu_2_O crystals. As shell thickness is decreasing, intensity of the Au peaks was getting stronger. Figure 1b was the SEM results of the samples, which displayed that the Au@Cu_2_O particles were uniformed cuboctahedrons with good monodispersity. TEM image (Fig. 1c) confirmed the formation of core-shell structure, and each cuboctahedron contained one Au TN. TEM image, size distribution histograms, and absorption spectrum of the pure Au TNs were showed in Additional file 1: Figure S1. The HRTEM image of the square regions illustrated the interplanar spacing of 2.13 and 2.48 Å, assigned to the spacing of the (2 0 0) and (1 1 1) plane of Cu_2_O. Moreover, the line scanning (Fig. 1e) of a single Au@Cu_2_O particle showed that gold element is principally located in the center of the particle while copper was bordered, which agreed well with the scanning transmission electron microscopy (STEM) (Fig. 1d) image.

### Size Control and Growth Process Study

**Fig. 2:**
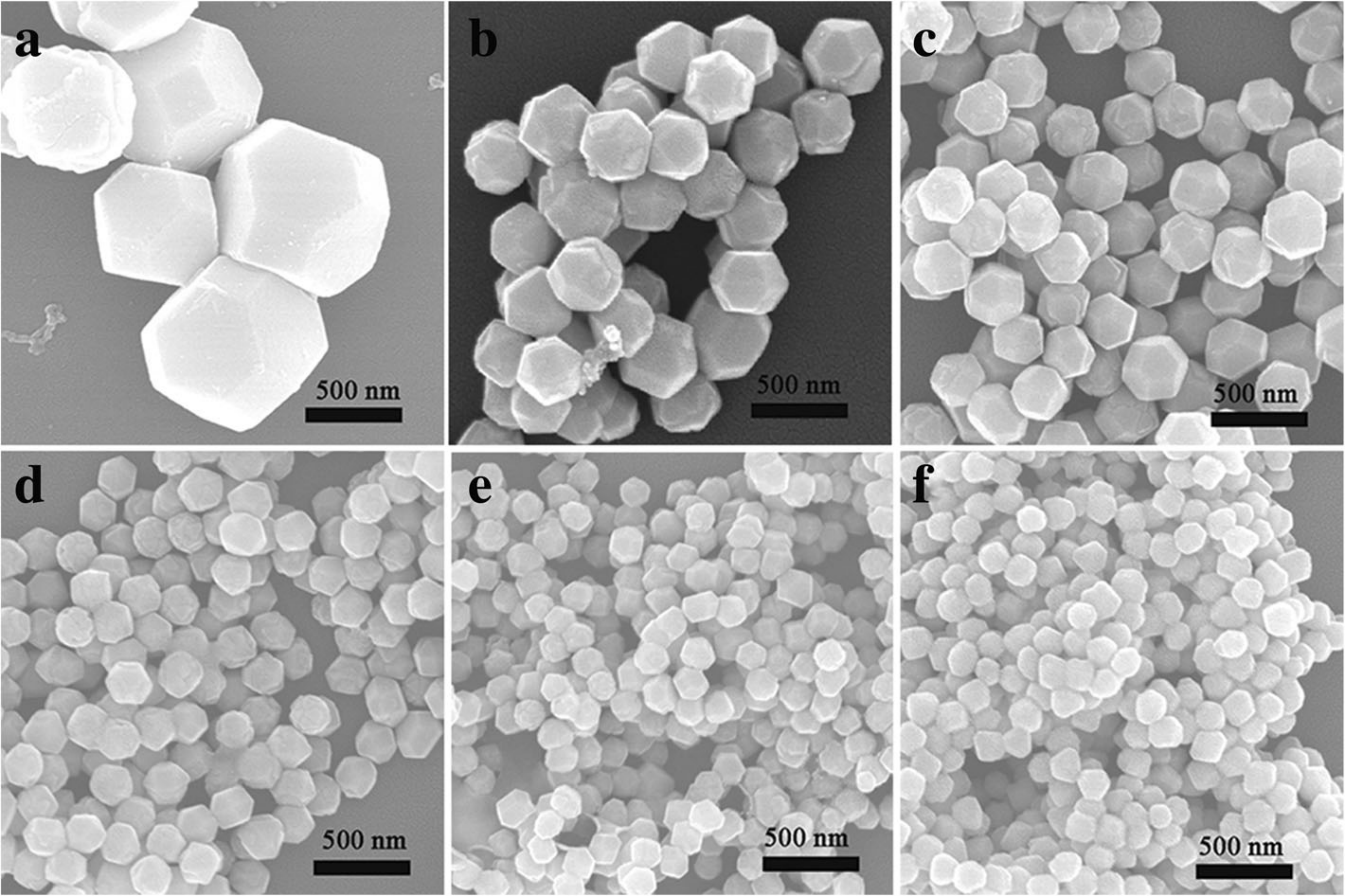
SEM images of the samples synthesized by adding (**a**) 0 ml (**b**) 0.1 ml (**c**) 0.2 ml (**d**) 0.4 ml synthesized by adding (**e**) 0.8 ml (**f**) 1.6 ml of gold core solution

SEM images of the samples (Fig. 2a–f) showed that particles with six different sizes were synthesized by adding 0, 0.1, 0.2, 0.4, 0.8, and 1.6 ml of Au core solution, respectively. Accordingly, the average particle sizes of the samples were estimated to be 555 nm, 349 nm, 281 nm, 225 nm, 176 nm, and 137 nm, respectively.Considering the average particle size of the Au TN was 31 nm (Additional file 1: Figure S1b), the average shell thickness of the samples was 159, 125, 97, 72.5, and 53 nm, respectively. Therefore, accurate size control could be realized, due to the fact that each Au@Cu_2_O core-shell structure contained one Au core [25]. As a result, a simple method was provided to adjust the thickness of Cu_2_O shell by altering the amount of the Au core in the synthetic process. The Au TN employed in the synthetic process was from the same batch, so the concentration of the seeds was invariable.

**Fig. 3:**
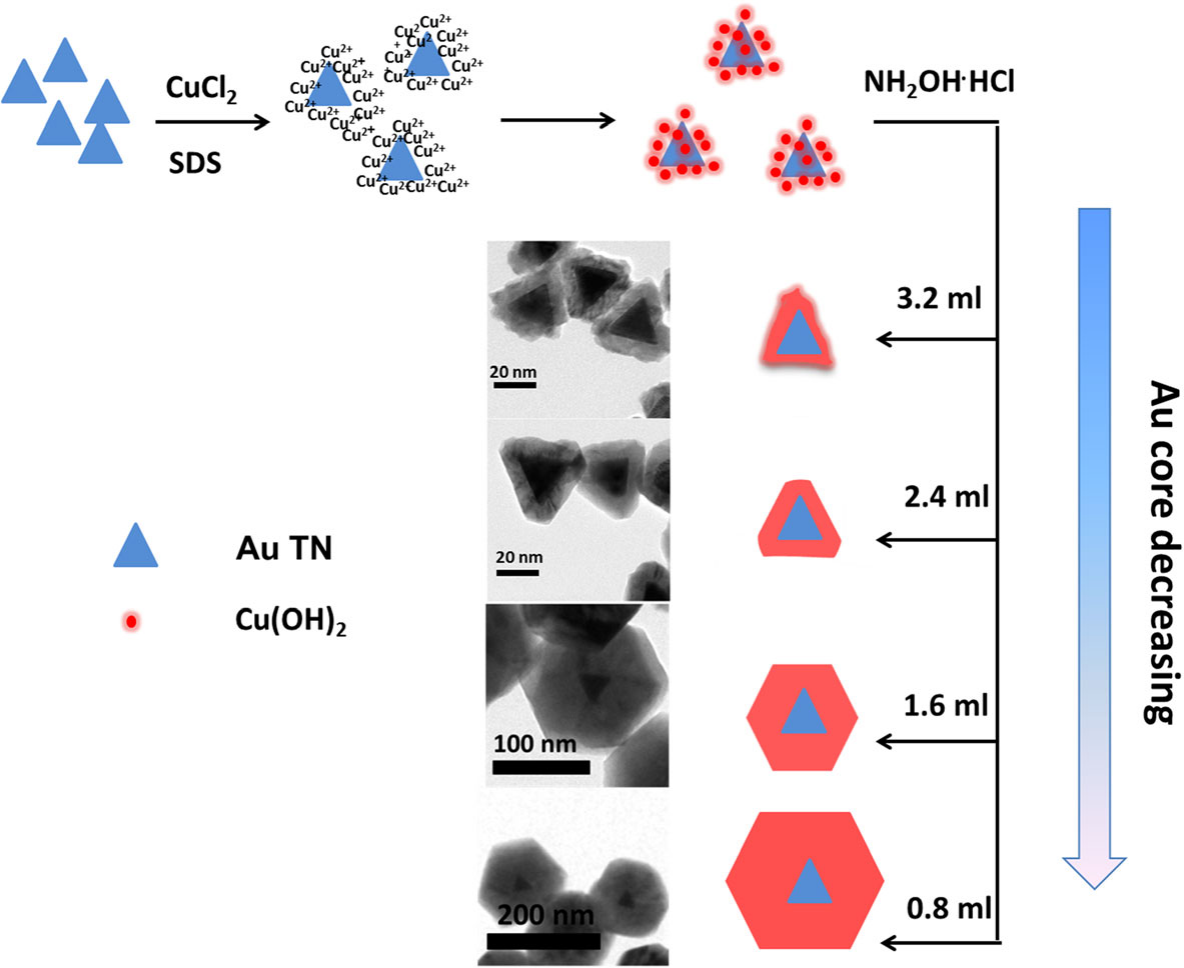
Schematic presentation for the growth process of the Au@Cu_2_O particles

To explore the growth process of the samples, a series of experiments had been done. When 3.2 ml of Au solution was added, particles with rough sheet structure were synthesized (as shown in Fig. 3), instead of expected cuboctahedron smaller than 53-nm Au@Cu_2_O, because Au cores were too much to capture enough Cu_2_O particles for the growth of complete cuboctahedron shell. When 2.4 ml of Au TN was added, every Au core was provided with more Cu_2_O particles. And the intermediate structure was synthesized because more Cu_2_O particles could adsorb on the surfaces of Au TN core. When the volume of Au TN was decreased to 1.6 ml, complete cuboctahedron was synthesized. As the volume of Au TN sequentially decreased, the morphology of the Au@Cu_2_O core-shell structure was maintained as cuboctahedron with particle size increasing.

### UV-vis and BET Analysis

**Fig. 4:**
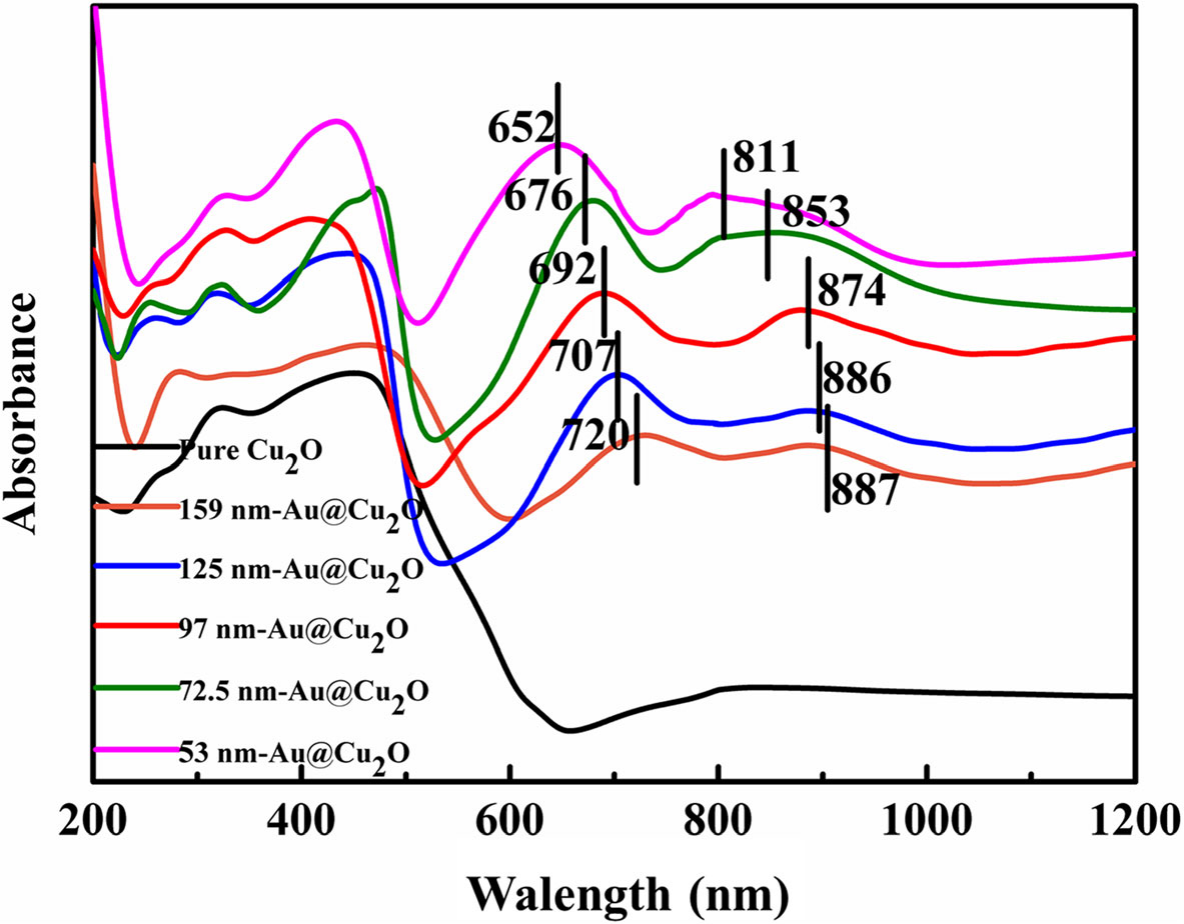
UV-vis diffuse reflectance spectra of the samples

Figure 4 represented the diffuse reflectance spectra of the samples. As the photograph showed (Additional file 1: Figure S2), the color of the reaction hydrosol was changing from dark orange to light green and then dark green. The distinct color change was obviously related to the core-shell interactions between Au TN core and Cu_2_O shell. Compared with the pure Cu_2_O, Au@Cu_2_O core-shell structure displayed strong absorption ability in the visible-light region. The absorption spectra at wavelength coverage below 500 nm were caused by the excitation interband transition of Cu_2_O shell. And the spectra at the wavelength coverage beyond 600 nm were attributed to the plasmon resonance of Au TN. In addition, with the shell thickness of Cu_2_O decreased, the plasmon peaks were blue-shifted drastically. The distinct blue-shift of the plasmon peak was because of the high refractive index and dielectric constant of the Cu_2_O shell. The plasmon resonance frequency of the noble metal was sensitive to the surrounding dielectric environment; therefore, the decrease of Cu_2_O shell thickness would cause blue-shift of the plasmon peak. Thus, Au@Cu_2_O core-shell heterostructure effectively adjusted the light absorption region of pure Cu_2_O and enhanced the visible-light absorption intensity.

**Fig. 5:**
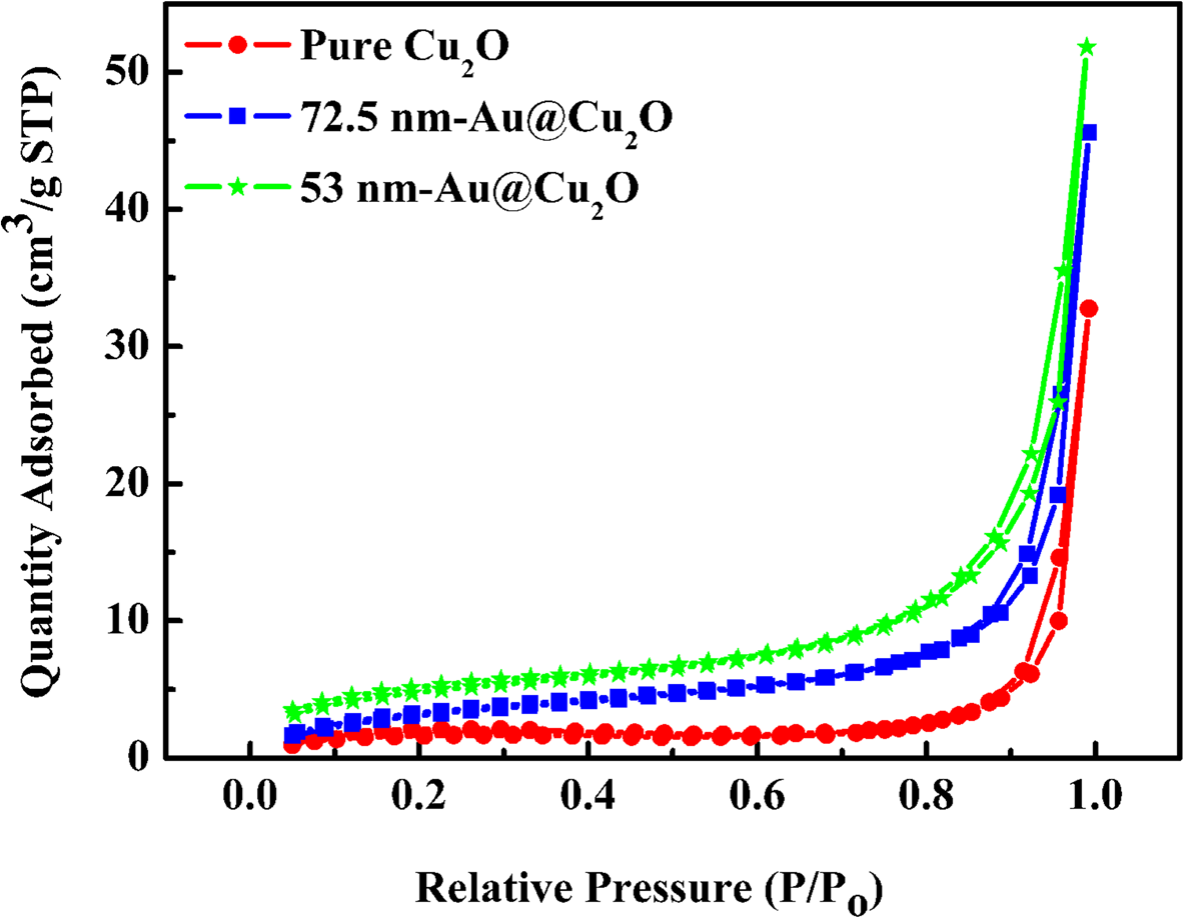
N_2_ adsorption-desorption isotherms of the samples

Nitrogen adsorption-desorption isotherms from the Brunauer*-*Emmett*-*Teller (BET) test showed (Fig. 5) that the specific surface areas of pure Cu_2_O, 72.5-nm Au@Cu_2_O and 53-nm Au@Cu_2_O were 7.00, 13.10, and 17.72 m^2^/g, respectively. This result demonstrated that the Au@Cu_2_O particles had similar specific surface areas.

### Photocatalytic Activity and Possible Mechanism

**Fig. 6:**
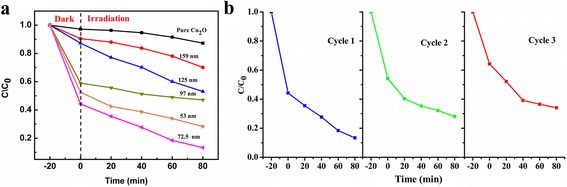
**a** Degradation curves of MO under visible-light irradiation. **b** Recyclability of the 72.5-nm Au@Cu_2_O for MO degradation

Figure 6a showed the degradation plots of MO involving photocatalytic activities of the samples. Compared with the pure Cu_2_O, Au@Cu_2_O particles showed better photocatalytic performance. As the shell thickness of the Au@Cu_2_O particles decreases, photocatalytic property was getting better. However, when the shell thickness was deceased from 72.5 to 53 nm, the photocatalytic property turned out to be worse. Thus, the 72.5-nm Au@Cu_2_O was found to exhibit the best catalytic performance, and after 80 min of the photoreaction, more than 86 % of MO was degraded.

The stability of the 72.5-nm Au@Cu_2_O was researched by degrading MO repeatedly, and the result was showed in Fig. 6b. The catalytic activity decreased slightly after each cycle, and considering the possible loss of catalysts during centrifugation, the stability was acceptable.

**Fig. 7:**
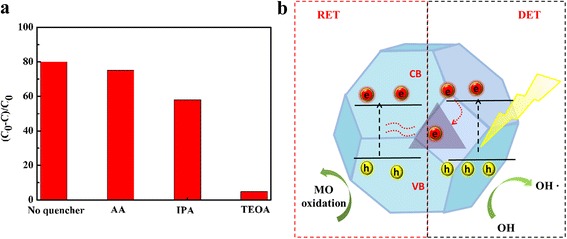
**a** Trapping experiment of the active species. **b** Schematic presentation for electron-transfer process of Au@Cu_2_O core-shell structure

To investigate the roles that different active species played in the photocatalytic process, IPA, TEOA, and AA were chosen as scavengers of ·OH, h^+^, and ·O_2_^−^ in the trapping experiment. Figure 7a showed that after the addition of three scavengers, the degradation rates of MO were reduced from 80 to 75.1, 58, and 4.8 %, respectively. This result illustrated that ·OH and h^+^ were the main active species in the photocatalytic process, and ·O_2_^−^ had little effect on the photocatalytic activity.

Based on the above analyses, the electron generation and transfer processes of charge carriers in the Au@Cu_2_O particles were displayed in Fig. 7b. On one hand, the Schottky barrier formed at the metal and semiconductor interface functioned as electron sink to capture and shift photoexcited electrons from the Cu_2_O shell to the Au TN. The process was considered as direct electron transfer (DET) [26], which effectively reduced the recombination rate of electron-hole pairs and prolonged lifetime of the holes. On the other hand, the near-field electromagnetic coupling between the LSPR dipole of Au TN and the interband transition dipole of Cu_2_O caused plasmon-induced resonant energy transfer (PIRET) [27], which effectively promoted charge separation in the Cu_2_O shell. Therefore, the improved catalytic activity might be attributed to the synergetic effect of the DET and PIRET.

## Conclusions

In summary, we have s Au@Cu_2_O core-shell structure with gold TN core and Cu_2_O shell. Moreover, the shell thickness of the core-shell particles could be controlled from 159 to 53 nm by adjusting the volume of Au solution. Photocatalytic experiment showed that Au@Cu_2_O core-shell structure exhibited better photocatalytic property than that of pure Cu_2_O, and the highest photocatalytic activity could be achieved on 72.5-nm Au@Cu_2_O which was 6.9 times better than that of pure Cu_2_O. The improved photocatalytic property was due to the integrated effect of the enhanced visible-light absorption and high separation rate of electron-hole pairs. This work would raise the possibility for exploring new core-shell structures and promote to probe more inspiring applications, apart from the field of photocatalysis, such as sensor or solar cell.

## Additional file

Additional file 1: Figure S1. (a) TEM image. (b) Particle size histograms and UV-is spectra of the Au triangular nanoplate (TN) colloids. **Figure S2.** Photograph of colloidal suspensions of the samples. (DOCX 443 kb)
