# Supplementary material for: Size Control and Growth Process Study of Au@Cu2O Particles
Source: Nanoscale Res Lett. 2016 Sep 8;11(1):390. doi: 10.1186/s11671-016-1603-6 (PMC5016315; doi:10.1186/s11671-016-1603-6)
Supplement: Additional file 1: Figure S1. — (a) TEM image. (b) Particle size histograms and UV-is spectra of the Au triangular nanoplate (TN) colloids. Figure S2. Photograph of colloidal suspensions of the samples. (DOCX 443 kb) [file 11671_2016_1603_MOESM1_ESM.docx]

**Supporting Information**

**Size control and growth process study of Au@Cu_2_O particles**

**Yuyuan Wang^a^, Min Zheng^b^, Shengnan Liu^a^, Zuoshan Wang^a^ ***

*a College of Chemistry, Chemical Engineering and Materials Science, Soochow University, Soochow, 215123,China*

*b College of Textile and Clothing Engineering, Soochow University, Soochow, 215123,China*

* Corresponding author. Fax: +86-0512-85187680; Tel: +86-0512-85187680

E-mail: [zuoshanwang@suda.edu.cn](mailto:zuoshanwang@suda.edu.cn);


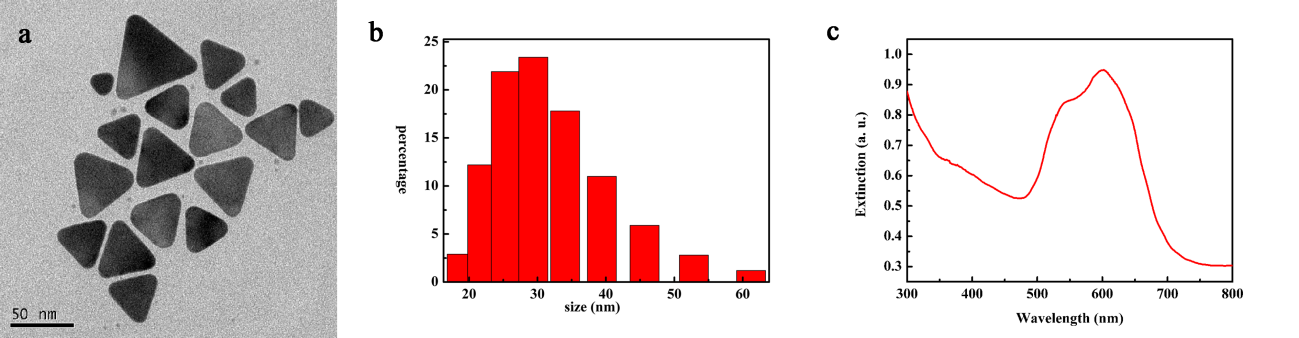


**Fig. S1** (a) TEM image (b) Particle size histograms and UV-is spectra of the Au triangular nanoplate (TN) colloids


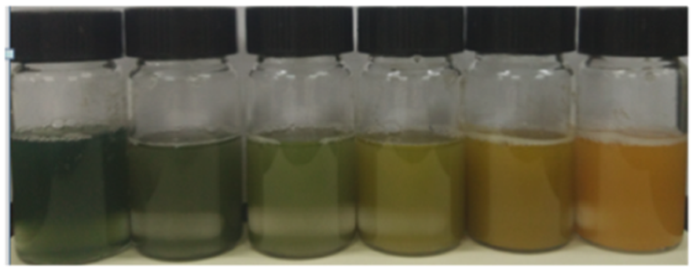


**Fig. S2** Photograph of colloidal suspensions of the samples
